# Anaphylaxis to epoxy resin during dental treatment

**DOI:** 10.1111/ddg.15850

**Published:** 2025-09-19

**Authors:** Julia Oberschmied, Elsbeth Oestmann, Margitta Worm

**Affiliations:** ^1^ Department of Dermatology Venereology and Allergy Charité – Universitätsmedizin Berlin, Germany

Dear Editors,

Allergic reactions to epoxy resins typically present as allergic contact dermatitis or airborne contact dermatitis.^1^, ^2^ Although rare, a few cases of immediate allergic reactions have been reported.^3^

We report on a 43‐year‐old male patient presenting for an allergological assessment following generalized urticaria, lip swelling, throat tightness and dyspnea during a root canal treatment. The acute anaphylactic reaction required emergency hospitalization. During the dental procedure, a local anesthetic containing articaine was administered, and AH Plus Jet® paste – an epoxy‐amine polymer‐based root canal filling material containing bisphenol A diglycidyl ether – was used.

The patient had experienced similar but milder episodes in previous dental treatments and reported an occupational exposure to epoxy resins about 20 years ago, which had caused pruritic skin lesions. Although he had had to change his occupation, no allergy testing was conducted at the time. There were no known allergies, but a history of childhood bronchial asthma and arterial hypertension treated with candesartan.

Allergological assessment included a skin prick testing with local anesthetics (procaine, lidocaine, bupivacaine, prilocaine, articaine, mepivacaine), which were all negative. A 20‐minute closed patch test was conducted using epoxy resin (based on bisphenol A diglycidyl ether) from the standard series and the synthetic resin adhesive series of the *German Contact Allergy Group*. The patch test resulted in a strong urticarial skin reaction at the sites corresponding to epoxy resin, 1,4‐butanediol diglycidyl ether, 1,6‐hexanediol diglycidyl ether, and butyl glycidyl ether (Figure 1). No delayed reactions were observed.

**FIGURE 1:**
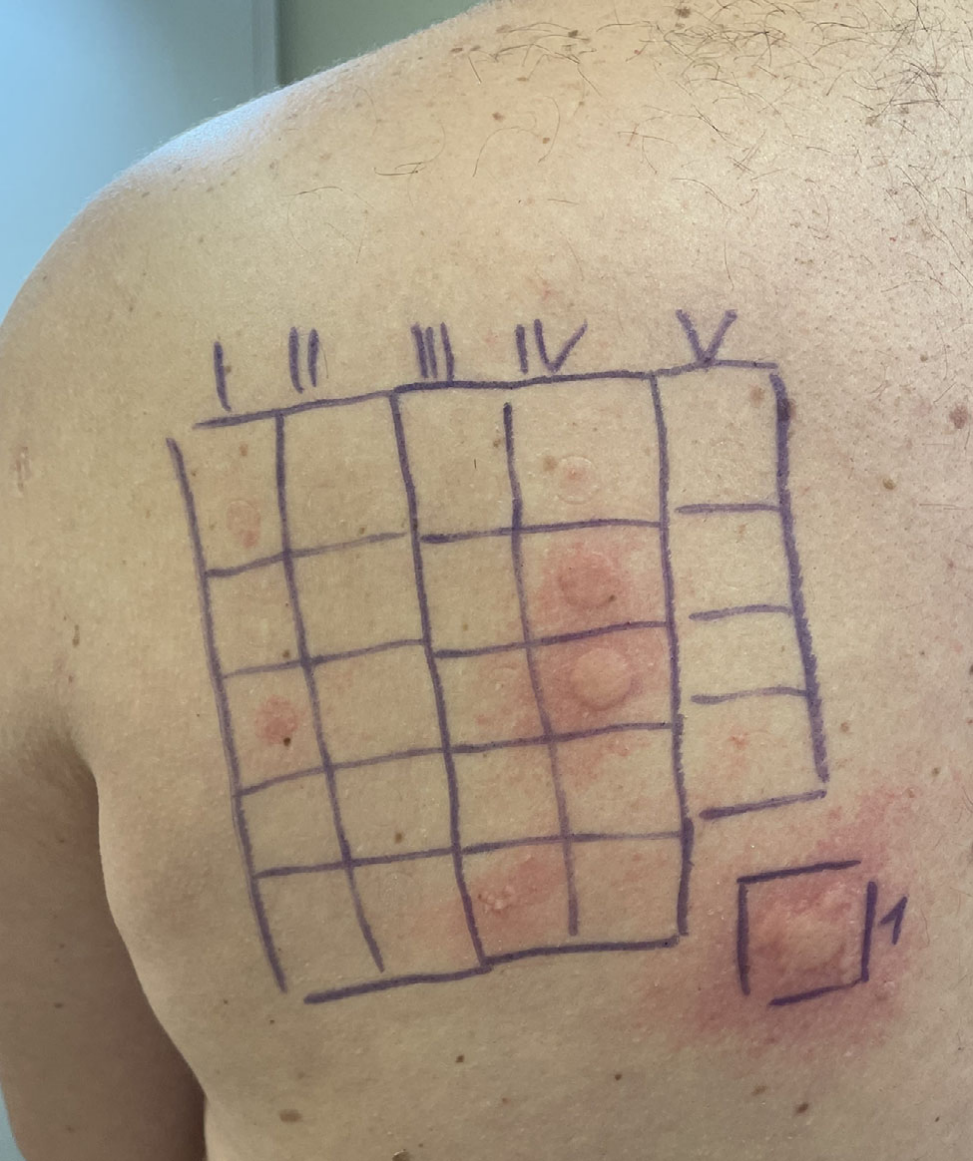
Positive immediate patch test reactions against epoxy resin (number 1), butyl glycidyl ether (row III, position five), 1,4‐butanediol diglycidyl ether (row IV, position two) and 1,6‐hexanediol diglycidyl ether (row IV, position three). The erythema in the first row (positions one and three) was considered irritative.

A Prick test for environmental allergens was positive for birch, grass, and ragweed. Total serum IgE was elevated at 229 kU/l, while specific IgE testing for latex was negative and the differential blood count normal.

In summary we identified epoxy resin based on bisphenol A diglycidyl ether as the cause of the anaphylactic reaction. Epoxy resins are widely used in various applications, including adhesives, plastics, floor coverings, insulating materials, paints, varnishes, and dental fillings. The term “epoxy resin” generally refers to an epoxy resin system composed of the resin itself, a hardener, reactive diluents, and other additives, all of which possess allergenic potential.^4^, ^5^ Approximately 75% of the epoxy resins currently in use are derived from bisphenol A diglycidyl ether (BADGE), which is also the most significant sensitizer.^6^ Epoxy resin has become an increasingly important contact allergen due to its widespread use, with the most common manifestations being allergic contact dermatitis and airborne contact dermatitis.^1^, ^2^, ^6^

Sensitization to epoxy resins is mostly occupational, occurring through direct skin contact, aerogenic dispersion, contaminated materials, inadequate protective equipment, or exposure to uncured epoxy resin.^7^ Hardened epoxy resins are generally considered harmless, as they neither sensitize nor provoke allergic reactions.^5^

As opposed to type IV sensitization causing eczema, only a few cases of type I allergies to epoxy resin systems have been described, primarily associated with contact urticaria, and less commonly with allergic rhinitis and occupational asthma.^3^, ^4^, ^8^ To date, only one case of an anaphylactic reaction to epoxy resin has been documented in the literature.^9^ To our knowledge, ours is the second case to be reported on anaphylaxis caused by a reaction to bisphenol A diglycidyl ether‐based epoxy resin. Furthermore, no reported cases of anaphylactic reactions to epoxy resin have yet been recorded in the *Anaphylaxis Registry*.

Our diagnosis was based on the clinical symptoms and the positive 20‐minute patch test, which indicated a contact urticaria, suggesting an IgE‐mediated reaction. Given the patient's history of an immediate systemic reaction, the urticarial response observed in the patch test, and the unavailability of the individual components, no further testing was performed.

As suggested previously,^9^ the reaction in our highly sensitized patient was caused by direct contact of the allergen with the mucous membranes during the dental procedure. We suspect that the initial sensitization occurred during his previous work, when he developed pruritic skin lesions after occupational epoxy resin exposure.

Our case highlights the importance of considering rare allergens, such as epoxy resins, in the differential diagnosis of anaphylaxis, particularly in patients with a history of occupational exposure and previous allergic reactions. Based on the data from the *Anaphylaxis Registry*, the most common triggers of occupational anaphylaxis are insects, followed by food and drugs.^10^

However, known type IV allergens (including fragrances and preservatives) can also trigger immediate reactions in some sensitized individuals.^11^ Therefore, if an immediate reaction is suspected based on the patient's medical history, the patch test should be read after 20 minutes and discontinued if strongly positive reactions occur.

## CONFLICT OF INTEREST STATEMENT

None
